# Co‐infection of coronavirus disease 19 and cytomegalovirus in a kidney transplant recipient

**DOI:** 10.1002/ccr3.7046

**Published:** 2023-03-04

**Authors:** Mehrdad Farrokhnia, Amir Baniasad, Fatemeh Mousavi Mehdiabadi

**Affiliations:** ^1^ Research Center of Tropical and Infectious Diseases Kerman University of Medical Sciences Kerman Iran; ^2^ Endocrinology and Metabolism Research Center, Institute of Basic and Clinical Physiology Sciences Kerman University of Medical Sciences Kerman Kerman Iran

**Keywords:** COVID‐19, cytomegalovirus, ESRD, kidney transplant

## Abstract

It has been suggested that severe SARS‐CoV‐2 infection could be a risk factor for Herpesviridae reactivation due to the state of sepsis‐associated immunosuppression. We presented the case of co‐infection of CMV and COVID‐19 infection in a 43‐year‐old woman with end‐stage renal disease.

## Abstract 2

Few reports suggested CMV reactivation in the cases of SARS‐CoV‐2 infection; most of them had several comorbidities and were critically ill. It remains to be determined if SARS‐CoV‐2 infection could be a risk factor for Herpesviridae reactivation, especially in immune‐compromised patients and if this co‐infection results in a worse prognosis.

## 1 INTRODUCTION

Coronavirus disease 2019 (COVID‐19) is a novel viral disease with an increasing number of cases worldwide. Solid organ transplant recipients are at a higher risk for COVID‐19 infection due to chronic immunosuppressants and comorbidities such as diabetes mellitus. Immune‐compromised patients may present atypically, and several reports of more than one infectious process are presenting simultaneously. Few previous reports of CMV and simultaneous SARS‐CoV‐2 infection suggested that severe SARS‐CoV‐2 infection could be a risk factor for Herpesviridae reactivation due to the state of sepsis‐associated immunosuppression.^1^

However, it has not been determined if immune‐compromised patients, such as solid organ transplant recipients, are at a higher risk of this co‐infection. Herein, we present the case of co‐infection of CMV and COVID‐19 infection in a kidney transplant recipient.

## 2 CASE HISTORY

A 43‐year‐old woman with end‐stage renal disease due to nephrolithiasis underwent a deceased‐donor kidney transplant in 2018 after 18 months of hemodialysis. At the time of transplantation, she received induction immunosuppression of thyroglobulin (ATG Fresenius, rabbit Anti‐thymocyte‐globulin) 6 mg/kg and methylprednisolone. Her immunosuppressive maintenance regimen included tacrolimus (3 mg daily in two divided doses with trough levels between 6 and 8 ng/mL), Mycophenolate mofetil (1500 mg a day), and prednisone (tapered to 5 mg). Valganciclovir (450 mg daily) was prescribed for CMV prophylaxis of CMV reactivation in the first 3 months after transplantation. She was seropositive for CMV; however, the CMV status of the donor was not known at the time of transplantation.

The immediate postoperative period was unremarkable, and she did not experience delayed graft function. She had a good function graft with a serum creatinine level of 0.9 mg/dL and an estimated glomerular filtration rate (eGFR) of 78 mL/min during the 20‐month follow‐up.

She was admitted to a primary rural health house on July 2020 with a 3‐day history of fever (38.2°C), malaise, and vomiting. She reported decreased urine output with no other symptoms. Also, she did not have a history of traveling or exposure to patients suspected of COVID‐19 infection.

On laboratory tests, white blood cell (WBC) count was 4.3 × 10^3^ μg/L, C‐reactive protein (CRP) of 9.5 mg/dL (normal range less than 6 mg/L), and serum creatinine levels of 1.3 mg/dL (eGFR 51 mL/min). She was hydrated, received viral gastroenteritis treatment, and was discharged within 48 hr with a serum creatinine of 1 mg/dL, with recommendations for oral hydration and on‐demand acetaminophen.

Five days later, the patient was admitted in a secondary healthcare center with persistent fever, nausea, vomiting, watery diarrhea, and decreased urine volume. Physical examinations revealed a body temperature of 38.1°C, blood pressure of 90/55 mm Hg, a pulse of 106 beats per minute, respiratory rate of 17 breaths per minute, and blood oxygen saturation of 96% on room air.

She was dehydrated. Lungs were clear, and no murmurs, rubs, or gallops were indicated during the heart examination. Her abdomen was soft and not tender, no organomegaly was detected, and the neurologic examination was unremarkable.

Laboratory parameters at the time of admission were as follows: blood glucose: 85 mg/dL, urea: 62 mg/dL, serum creatinine: 1.9 mg/dL (eGFR of 32 cc/min), plasma sodium: 135 meq/L, plasma potassium: 5 meq/L, calcium: 8.3 mg/dL, phosphorus: 2.3 mg/dL, magnesium: 1.8 mg/dL and serum uric acid of 9.2 mg/dL.

AST: 101 U/L, ALT: 90 U/L, CRP: 18.3 mg/L(normal range of <6 mg/L), ESR: 43 mm/h, WBC count: 2.8 × 10^3^ μg/L, hemoglobin: 9.9 g/dL, and platelet count: 105 × 10^3^ μg/L. Coagulation parameters were within normal reference values. Urinalysis revealed 2–3WBC, 8‐10RBC, protein (+), and nitrites (−).stool examination revealed 1–2 WBC and no RBC.

The Ultrasonographical study revealed unremarkable findings in the allograft.

The patient was admitted, and once blood cultures had been taken, ciprofloxacin (200 mg bid IV) was administered on an empirical basis. High‐resolution computed tomography (HRCT) of the chest revealed no lung abnormalities.

Nasopharyngeal swab specimens were collected for testing COVID‐19 (RT‐PCR assay) which was reported positive. The trough level of tacrolimus was sent, which reported 21 ng/mL.

MMF was stopped, and the tacrolimus dose was reduced to 1 mg BID. During hospitalization, the corticosteroid dose was increased to 15 mg daily.

The patient was observed for hydration status, urine output, oxygen saturation, and respiratory symptoms. After 5 days of admission, the patient did not feel better regarding diarrheal defecation, abdominal discomfort, malaise, and anorexia, while serum creatinine was 2.1 mg/dL. Serum was sent for CMV polymerase chain reaction (PCR), which showed the presence of 38,000 viral copies/ml. Ganciclovir was prescribed parenterally. The frequency of diarrheal defecation decreased, the patient became afebrile, and oral intake was well tolerated within a week. Considering the established diagnosis of CMV infection in our patient based on viral load, there was no need to confirm the diagnosis. So, the colonoscopy did not perform for the patient.

The obtained blood culture was negative, and serum creatinine levels decreased to 1.5 mg/dL. The patient was discharged on Day 16 after admission with oral Valganciclovir (900 mg bid) and prednisolone (10 mg daily), and tacrolimus (1 mg bid).

After 2 weeks of outpatient follow‐up, the patient was afebrile with no respiratory symptoms, but she suffered from anorexia and loose stool. Blood oxygen saturation levels were 97% on room air. Serum creatinine level was 1.3 mg/dL, blood WBC count 3.5 × 10^3^ μg/L, platelet count 98 × 10^3^ μg/L, liver enzymes were within the normal range, CRP 8 mg/L (normal range of <6 mg/L) and tacrolimus trough level of 14 ng/mL. Tacrolimus was replaced by cyclosporine (50 mg bid), and oral Valganciclovir was continued with a dose of 900 mg daily.

In her 2‐week follow‐up, she felt better with no gastrointestinal symptoms. Serum creatinine was 0.9 mg/dL, blood leukocyte count of 4.2 × 98 × 10^3^ μg/L, platelet count 156× 10^3^ μg/L, plasma cyclosporine level (C2) of 440 ng/mL, and negative qualitative CRP.

After 2 weeks of treatment, CMV PCR was rechecked, which was negative. Prophylactic oral Valganciclovir was prescribed for the following 2 months (450 mg bid), and MMF (1.5 gr/day) was added to cyclosporine and prednisolone (5 mg/day).

## 3 DISCUSSION

COVID‐19, a novel respiratory viral disease first identified in late December 2019, emerged in Wuhan, China. This viral outbreak resulted in almost 105 million recognized infections worldwide at the time of writing this article.

Based on the data released in February 2021 by the World Health Organization, as reported by Iranian national authorities, the total number of confirmed cases is almost a 1.5 million.

Solid organ transplant recipients (SOTRs) may be at a higher risk of developing critical COVID‐19 illness due to chronic immunosuppression and comorbidities.

International registries demonstrated that SOTRs are at higher risk of early mortality in hospitalized patients with COVID‐19 infection. However, it is unclear if immunosuppressive treatment is an independent risk factor for mortality, considering all the other comorbidities such as age, gender, obesity, diabetes, and hypertension.^2^, ^3^, ^4^

Multiple factors, including reduced renal perfusion due to a reduction in effective arterial circulating volume, acute tubular injury from cytokine storm, and multi‐organ failure, may contribute to acute kidney damage in patients with COVID‐19. Also, kidney transplant recipients are at higher risk of these damages.

Patients affected by SARS‐CoV‐2 have a spectrum of diseases ranging from asymptomatic or mild illnesses to severe conditions with acute respiratory distress syndrome.^5^ Few cases of mild COVID‐19 infection have been reported in kidney transplant recipients.^6^, ^7^

Atypical clinical manifestations such as an unspecified viral infection or gastrointestinal symptoms have been reported in renal transplant recipients.^8^

COVID‐19 infections may be associated with gastrointestinal symptoms because of their affinity to the angiotensin‐converting enzyme 2 (ACE‐2) and to the transmembrane protease serine‐2, expressed in the lung and gastrointestinal tract.

It has been suggested that patients with COVID‐19 infection limited to the gastrointestinal system have a more benign course and a lower mortality rate.

In the registry reported by Crespo et al., about a third of their registry population (509 KT recipients with COVID‐19) presented with gastrointestinal symptoms, and they survived over 40% more than KT recipients without digestive symptoms.^9^

There are several reports of CMV reactivation in renal transplant recipients, typically within the first months after transplantation. The most definitive risk factor of CMV reactivation is the serological incompatibility between donor and recipient, especially when a seropositive donor donates an organ to a seronegative recipient. Late CMV reactivation (after >6 months of transplantation) is not so prevalent and often related to the need for increased immunosuppression because of late graft rejection.

The most striking feature of this case is the late reactivation of CMV after about 2 years of renal transplantation, while she received maintenance therapy for 2 years and had an excellent graft function.

It has been recognized that viral infections may be followed by a cascade of complex interactions with the host defense system that can facilitate infections due to other viruses.

Few reports suggested CMV reactivation in the cases of SARS‐CoV‐2 infection; most of them had several comorbidities and were critically ill with severe lung involvement; it has been suggested that SARS‐CoV‐2 infection and its strong activation of the innate and cellular immune system may trigger CMV reactivation.

The first case of co‐infection with COVID‐19 and cytomegalovirus (CMV) was reported by D'Ardes et al.^10^

Amaral Pedro et al. reported a tissue‐invasive gastrointestinal CMV infection in a critically ill patient admitted with ARDS due to COVID‐19 infection, which was entirely recovered by ganciclovir.^11^

However, it needs to be clarified whether KTRs are at increased risk of these co‐infections or whether their clinical and renal outcomes will alter.

A case series by Molaei et al. reported co‐infection of CMV in 4 out of 10 KTRs infected with COVID‐19. They suggested the worse outcome for patients with these co‐infections.^12^

All 4 cases were diabetic, and 2 out of 4 were smokers. They all had respiratory symptoms and parenchymal lung involvement in the CT scans.

There is no report of the gastrointestinal phenotype of COVID‐19 and concurrent CMV infection in KTRs yet. Our patient was not diabetic and had no smoking history, and there were no clinical symptoms or radiological findings of lung involvement. The mild course of her disease may be attributed to the absence of severe comorbidity, or it may be the result of the enteric phenotype of her COVID‐19 disease.

It has been suggested that the stimulation of the innate immune response induced by other infections (named trained immunity) would favor the early synthesis of interferon and Toll‐like receptors, which could explain the benign course of COVID‐19 infection.^13^

In conclusion, this report indicates that it is essential not to ignore other pathogens in febrile syndromes during this pandemic. The reportable point of our case was that our patient was not diabetic or a smoker, and lungs were spared despite the co‐infection of CMV and SARS‐Cov‐2.

It remains to be determined if SARS‐CoV‐2 infection could be a risk factor for Herpesviridae reactivation, especially in immune‐compromised patients such as recipients of solid organs, and if this co‐infection necessarily results in a worse prognosis.

## AUTHOR CONTRIBUTIONS

**Mehrdad Farrokhnia:** Conceptualization; supervision; writing – review and editing. **Amir Baniasad:** Conceptualization; writing – original draft; writing – review and editing. **fatemeh mousavi mehdiabadi:** Conceptualization; project administration; supervision; writing – original draft; writing – review and editing.

## FUNDING INFORMATION

There is no external funding source for this case report.

## CONFLICT OF INTEREST STATEMENT

There is no conflict of interest.

## ETHICAL APPROVAL

The patient's informed consent has been obtained, and the “Iran National Committee has approved the therapy procedure for Ethics in Biomedical Research” (http://ethics.research.ac.ir/IndexEn.php, no: IR.KMU.REC.1401.120).

## CONSENT

Written informed consent was obtained from the patient to publish this report in accordance with the journal's patient consent policy.

## Data Availability

The corresponding author's data supporting this study's findings are available upon reasonable request.
